# Diversity of Matriptase Expression Level and Function in Breast Cancer

**DOI:** 10.1371/journal.pone.0034182

**Published:** 2012-04-13

**Authors:** Arkadiusz Welman, Duncan Sproul, Peter Mullen, Morwenna Muir, Andrew R. Kinnaird, David J. Harrison, Dana Faratian, Valerie G. Brunton, Margaret C. Frame

**Affiliations:** 1 Edinburgh Cancer Research Centre, Institute of Genetics and Molecular Medicine, University of Edinburgh, Edinburgh, Scotland, United Kingdom; 2 Edinburgh Breakthrough Research Unit and Division of Pathology, Institute of Genetics and Molecular Medicine, University of Edinburgh, Edinburgh, Scotland, United Kingdom; 3 Medical Research Council Human Genetics Unit, Institute of Genetics and Molecular Medicine, University of Edinburgh, Edinburgh, Scotland, United Kingdom; Sun Yat-sen University Medical School, China

## Abstract

Overexpression of matriptase has been reported in a variety of human cancers and is sufficient to trigger tumor formation in mice, but the importance of matriptase in breast cancer remains unclear. We analysed matriptase expression in 16 human breast cancer cell lines and in 107 primary breast tumors. The data revealed considerable diversity in the expression level of this protein indicating that the significance of matriptase may vary from case to case. Matriptase protein expression was correlated with HER2 expression and highest expression was seen in HER2-positive cell lines, indicating a potential role in this subgroup. Stable overexpression of matriptase in two breast cancer cell lines had different consequences. In MDA-MB-231 human breast carcinoma cells the only noted consequence of matriptase overexpression was modestly impaired growth in vivo. In contrast, overexpression of matriptase in 4T1 mouse breast carcinoma cells resulted in visible changes in morphology, actin staining and cell to cell contacts. This correlated with downregulation of the cell-cell adhesion molecule E-cadherin. These results suggest that the functions of matriptase in breast cancer are likely to be variable and cell context dependent.

## Introduction

Matriptase (also known as MT-SP1, ST14, TADG-15 and epithin) is a member of the family of type II transmembrane serine proteases [1]. It is an 80–90 kDa glycoprotein with complex structure, regulatory mechanisms and functions [2], [3]. It consists of a cytoplasmic N-terminus of unknown function, a short transmembrane part, and a large C-terminal region containing a catalytic serine-protease domain and several non-catalytic domains (a single SEA, two CUB and four LDLRA domains). Matriptase is synthesized as an inactive single-chain zymogen on the rough endoplasmic reticulum and travels to the plasma membrane via the Golgi apparatus [2]. The extracellular part of matriptase can also be shed from the cell surface into the surrounding microenvironment. The mechanisms that trigger the activation of matriptase as well as the details of the activation and the shedding processes remain incompletely understood. It is believed that full matriptase activation requires two sequential endoproteolitic cleavages and transient interaction with its cellular inhibitor HAI-1 [2], [4]. Recent evidence indicates that activation of matriptase can occur both on the cell surface and inside the cells and may be an early response to acidosis [5].

Matriptase is important for maintaining epithelial integrity and mice deficient in this protein die within 48h after birth due to compromised epidermal barrier function [6]. The spectrum of known matriptase substrates includes extracellular matrix proteins [3], [7], cell adhesion molecules [8], ion channels [9], growth-factor-like proteins [10], [11] and other proteases [12]. Its actions can result in protein processing, activation or degradation. Importantly, there is a large body of evidence implicating matriptase in tumour formation and metastasis [3], [7]. Even low level overexpression of matriptase is sufficient to trigger tumor formation in mice [13]. In addition, there is significant evidence linking matriptase to carcinogenesis in several cancer types including ovarian, prostate and cervical cancers [3], [14]. Consequently, there is considerable activity in the development of matriptase inhibitors [15], [16], [17], and methods to monitor matriptase activity in tumors [18], [19].

Although matriptase was originally discovered as a matrix-degrading protease in breast cancer cells [20], its significance and role(s) in breast cancer remain poorly understood. Hence, the validity of matriptase as a target in breast cancer therapy remains to be established. There are only a few published studies that have attempted to address the importance of matiptase in breast cancer and no robust conclusions have emerged (see discussion for further information). We analysed matriptase expression in 16 human breast cancer cell lines and in 107 primary breast tumors using reverse phase protein arrays. We also studied the consequences of overexpressing matriptase in two breast cancer cell lines. Our results show that although some cancer cell lines and primary tumors do express matriptase at relatively high levels, a significant proportion do not express matriptase at all, or at subdetectable levels. Matriptase expression was not significantly associated with node status, grade or tumor size. Morover, overexpression of matriptase in MDA-MB-231 and 4T1 cells had different phenotypic consequences implying that the function(s) of matriptase in breast cancer cells are variable.

## Results

### Expression of matriptase in breast cancer cell lines

**Figure 1:**
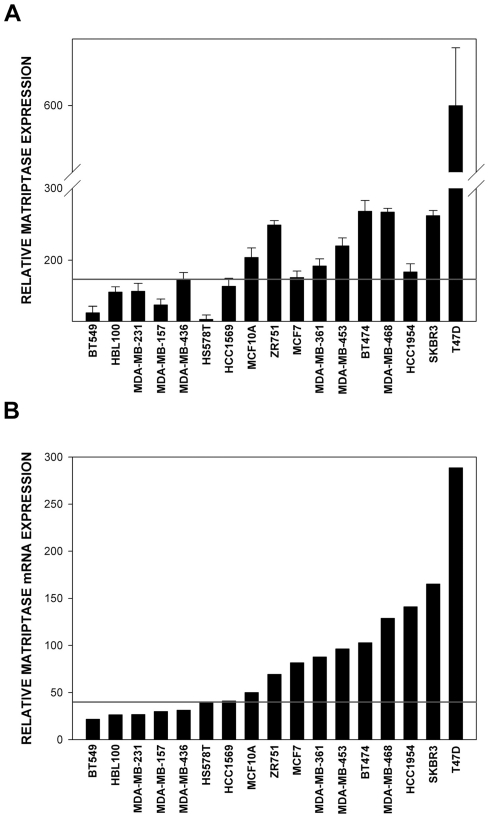
Analysis of matriptase mRNA and protein levels in a panel of 16 human breast cancer cell lines and a non-tumorigenic breast epithelial cell line MCF10A. (A) Matriptase protein levels in indicated cell lines as determined using reverse phase protein arrays (RPPA). (B) Matriptase mRNA expression levels in indicated cell lines based on the data from Array Express (E-TABM-157). The “cut off” line in (A) was set at the value registered for the MDA-MB-436 cells. These cells displayed the highest RPPA read-out from all the cell lines that showed no detectable matriptase expression as validated by western blots presented in Fig. S1. Therefore the line represents the highest registered background reading. The “cut off” for Matriptase mRNA expression levels shown in (B) was determined using statistical information from the expression arrays. For some cell lines the level of expression was called as not significant above background (or “Absent”). The “cut off” was set to the expression level of the highest “Absent” cell line (HCC1569). Error bars represent standard deviations.

High expression level of matriptase is a consistent feature of multiple human tumors of epithelial origin, but the amount of data available on the abundance of this protein in breast cancers remains relatively scarce. We analysed the expression of matriptase at the protein level in multiple established human breast cancer cell lines using reverse phase protein arrays (RPPA) [21]. To be sure that the RPPA data truthfully reflected matriptase expression levels in the samples, we independently confirmed them using standard western blotting approach with a different batch of antibody and a different signal detection system (see Materials and Methods for details). As shown in **Fig. 1A** and **Fig. S1** there was a very good correlation between the results obtained using RPPA and classical western blotting approach. Out of 16 breast cancer cell lines tested, seven did not express detectable levels of matriptase (∼44%; BT549, HBL100, MDA-MB-231, MDA-MB-157, MDA-MB-436, HS578T and HCC1569) and nine showed detectable expression of this protein (∼56%; ZR751, MCF7, MDA-MB-361, MDA-MB-453, BT474, MDA-MB-468, HCC1954, SKBR3 and T47D). There was significant diversity in the expression levels of matriptase among those breast cancer cell lines that did show detectable levels of the protein, with T47D cells displaying at least a few times higher level than any other cell line tested. Interestingly, the majority of the breast cancer cell lines with detectable matriptase expression showed higher levels of the protein than the non-tumorigenic breast epithelial cell line MCF10A used as a control [22], and the HER2 positive cell lines SKBR3, BT474, and T47D all showed high expression of matriptase.

The results described above demonstrated that breast cancer cell lines can be divided into two categories based on matriptase protein level: (i) those that display significant amounts of the protein, and (ii) those with no detectable matriptase protein expression. In an attempt to understand the reasons for this diversity we employed a bioinformatics-based approach. We analysed matriptase mRNA expression in the same 17 cell lines using raw data provided by Neve et all [23] and RMA algorithm combined with an updated microarray annotation [24]. As illustrated in **Fig. 1B**, all the cell lines that displayed undetectable matriptase protein levels also showed neglectable amounts of matriptase mRNA. This indicated that downregulation of matriptase in those cell lines was associated with pre-translational rather than post-translational mechanisms. There was no clear quantitative relationship between the amount of matriptase mRNA and matriptase protein levels in the remaining cell lines (compare **Fig. 1A** and **Fig. 1B**).

### Expression of matriptase in primary breast tumors

**Figure 2:**
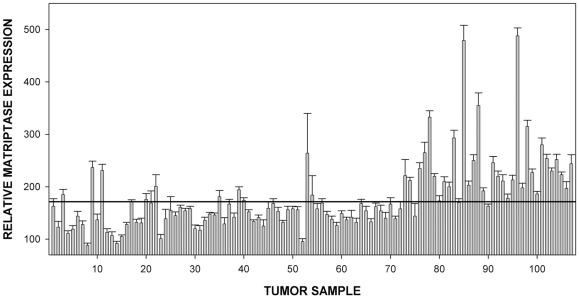
Analysis of matriptase protein levels in a panel of 107 primary tumor biopsies from Edinburgh Breast Cancer Unit. The samples were spotted on the same slides as cell lines in Fig. 1 and the “cut off” line is placed at identical value as in Fig. 1A. Error bars represent standard deviations.

The results obtained using breast cancer cell lines confirmed the utility of RPPA approach to determine matriptase protein levels. We also analysed matriptase expression in protein lysates from 107 primary breast tumor biopsies spotted on the same arrays as the cell line lysates. Patient details are shown in **Table S1**. Analysis of the data revealed that primary tumor samples displayed considerable diversity in matriptase protein level reflecting the variability observed among the cell lines (**Fig. 2**). There were no significant correlations between matriptase expression and tumor node status, grade or size (data not shown). However, matriptase expression measured was higher in HER2 expressing tumors compared to HER2 negative (immunohistochemical score 0) tumors (183.8 vs 157.0, p = 0.121, student's T-test). In order to further explore this association, HER2 expression was measured quantitatively using RPPA (data not shown). There was a good correlation between HER2 and matriptase expression (Spearman's Rho 0.57, p<0.001), indicating that matriptase expression is higher in HER2-positive tumors.

**Figure 3:**
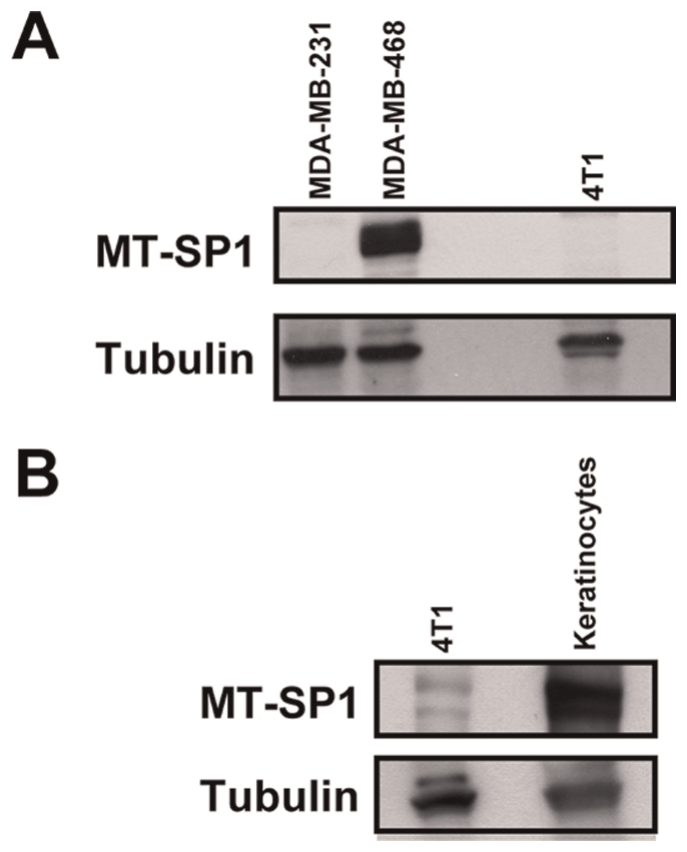
Matriptase (MT-SP1) protein levels in MDA-MB-231 and 4T1 cells as compared to human MDA-MB-468 cells (A), and primary mice keratinocytes (B). Tubulin represents the loading control.

### Consequences of matriptase overexpression in MDA-MB-231 human breast carcinoma and 4T1 mouse breast carcinoma cells in vitro

The data obtained using protein arrays indicated that expression of matriptase in breast cancers is rather heterogenous with some tumors displaying high levels of the protein, and others showing no detectable levels. This could imply that matriptase may have diverse functional consequences and its roles in breast tumorigenesis may depend on cellular context. We decided to investigate this possibility by overexpressing matriptase in two breast carcinoma cell lines with different characteristics: (i) MDA-MB-231 human breast carcinoma cells [25], and (ii) 4T1 mouse breast carcinoma cells [26]. The MDA-MB-231 cells represent a well characterised breast cancer model system and are part of the NCI-60 human cancer cell line panel. The 4T1 cells were selected as a second model because they show low level of endogenous matriptase (**Fig. 3**), and display epithelial rather than mesenchymal morphology. This is in contrast to available human breast cancer cell lines devoid of matriptase that display mesenchymal-type morphology [27].

**Figure 4:**
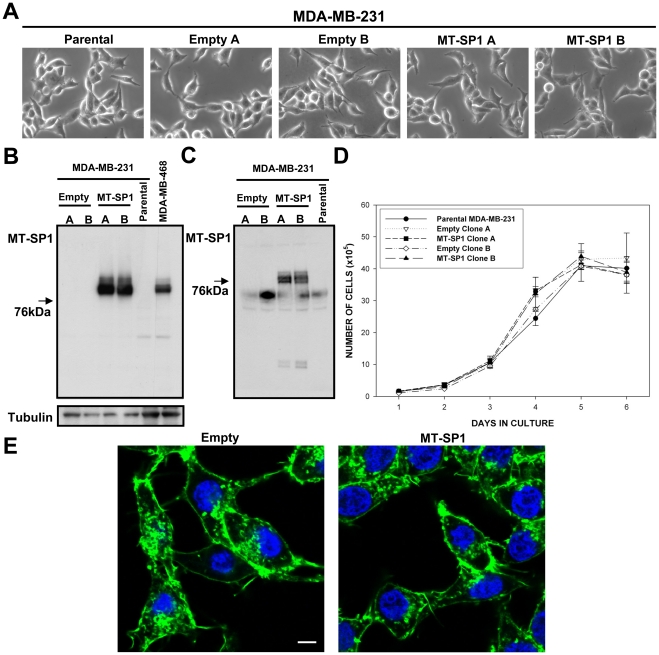
Properties of the MDA-MB-231 cells engineered to overexpress matriptase **(MT-SP1 A, MT-SP1 B) and the respective control clones (Empty A,** **Empty B).** (A) Bright field images of the selected clones and parental cells. (B) Western blots performed on total cell lysates, and (C) proteins precipitated from conditioned medium, using matriptase specific antibody. (D) In vitro growth curves for the selected clones and the parental cell line. There were no significant differences between the clones (p>0.05). (E) Representative fluorescence images of MT-SP1 B and Empty A cells stained with fluorescein-labelled phalloidin (actin cytoskeleton) and DAPI (nuclei). Similar results were obtained with MT-SP1 A and Empty B clones. Scale bar 10 µm.

**Figure 5:**
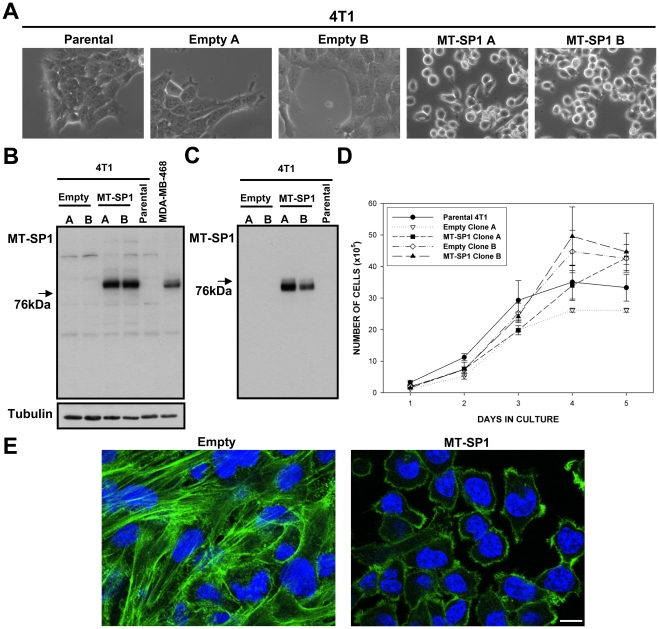
Properties of the 4T1 cells engineered to overexpress matriptase (MT-SP1 A, MT-SP1 B) and the respective control clones (Empty A, Empty B). (A) Bright field images of the selected clones and parental cells. (B) Western blots performed on total cell lysates, and (C) proteins precipitated from conditioned medium, using matriptase specific antibody. (D) In vitro growth curves for the selected clones and the parental cell line. Although some “between-clone” variations were found (p<0.05) they were not associated with the presence or absence of MT-SP1 overexpression. (E) Representative fluorescence images of MT-SP1 B and Empty B cells stained with fluorescein-labelled phalloidin (actin cytoskeleton) and DAPI (nuclei). Similar results were obtained with MT-SP1 A and Empty A clones respectively. Scale bar 10 µm.

As illustrated in **Fig. 4B** and **Fig. 5B** we were able to establish matriptase overexpressing clones in both cellular backgrounds. Importantly, matriptase in the established clones seemed to undergo expected trafficking and posttranslational processing as demonstrated by the fact that shed matriptase could be detected extracellulary in the conditioned medium from those cells (**Fig. 4C** and **Fig. 5C**).

Shedding and release of matriptase from cell surface into conditioned medium is a typical feature of breast cancer cells that naturally express this protease [28].

**Figure 6:**
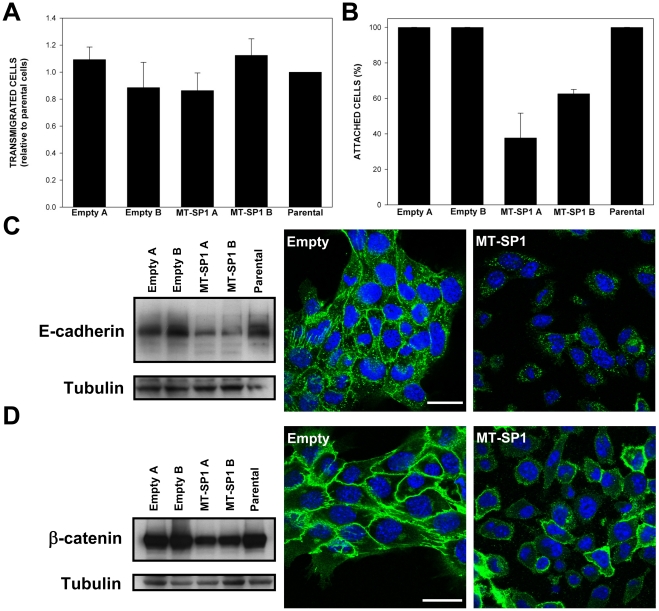
In vitro adhesive and migratory properties of selected 4T1 clones stably **overexpressing matriptase (MT-SP1) and respective control cells.** (A) Migratory properties of indicated cells as determined in the Transwell migration assay. No statistically significant differences between the clones were found (p>0.05). (B) Attachment strength of indicated clones as determined in the detachment assay. The results obtained for MT-SP1 overexpressing clones were significantly different (p<0.005) from those obtained for control clones and parental cell line. (C) Western blots illustrating E-cadherin expression in the indicated cell lines (left), and representative immunofluorescence pictures of E-cadherin staining (green) in MT-SP1 overexpressing cells and control cells (right). (D) Western blots illustrating β-catenin expression in the indicated cell lines (left), and representative immunofluorescence pictures of β-catenin staining (green) in MT-SP1 overexpressing cells and control cells (right). The immunofluorescence data in panels (C) and (D) are for clones 4T1 Empty B and 4T1 MT-SP1 B respectively, but analogous results were obtained in 4T1 Empty A and 4T1 MT-SP1 A clones. Blue color represents DAPI staining (nuclei). The individual (not overlayed) images for β-catenin and DAPI stainings presented in panel (D) are provided in Figure S4. Error bars in (A) and (B) represent standard errors. Scale bars 30 µm.

As shown in **Fig. 4**, even relatively high level overexpression of matriptase in MDA-MB-231 cells had no visible effects on cellular morphology (**Fig. 4A**) or actin cytoskeleton organisation (**Fig. 4E**). We also could not detect any matriptase-caused effects on in vitro growth, migration or adhesion in this cellular background (**Fig. 4D** **and Fig. S2**). In contrast, matriptase overexpression in 4T1 cells at a level comparable to that observed in human breast cancer cell lines (**Fig. 5B**), was sufficient to trigger obvious phenotypical changes. Distinct from cells transfected with the control plasmid, the majority of matriptase transfected 4T1 cells displayed a more rounded phenotype (**Fig. 5A**). Importantly, the maintenance of this rounded phenotype seemed to require continuous expression of high levels of matriptase because the cells that spontaneously reverted to a more flat morphology displayed significant reduction in matriptase overexpression level (**Fig. S3**). The rounded phenotype was associated with changes in the actin cytoskeleton and much less pronounced cell-cell contacts (**Fig. 5A, E**). We did not notice any consistent effects of matriptase overexpression on in vitro proliferation or migration of selected 4T1 cell clones (**Fig. 5D** **and** **Fig. 6A**), but the cells that overexpressed matriptase were much easier to detach from the tissue culture dish than their empty vector-transfected counterparts (**Fig. 6B**). This suggested that in the 4T1 cellular background matriptase overexpression may affect cell adhesion. To address this possibility we first analysed the status of the non-receptor tyrosine kinase c-Src and focal adhesion kinase (FAK) – two of the key regulators of actin dynamics and cell-extracellular matrix adhesions [29]. As shown in **Fig. S4**, although there were some differences in the intracellular distribution of FAK and phosphorylated form of Src between matriptase overexpressing clones and control clones (perhaps reflecting the observed differences in the organisation of the actin cytoskeleton), there were no significant differences in the total protein levels of FAK and c-Src, or in the levels of phosphorylation of these proteins at the major regulatory sites (P-FAK Y397 and P-Src Y416 respectively). We subsequently analysed the effects of matriptase overexpression on the key cell-cell adhesion molecule E-cadherin. E-cadherin controls epithelial morphology and is often lost or internalised during epithelial-mesenchymal transition, a process that has been implicated in tumor progression and metastasis [30]. As illustrated in **Fig. 6B** we found a reduction in E-cadherin protein levels in cells overexpressing matriptase as compared to control cells. In addition, immunofluorescence demonstrated that E-cadherin in matriptase overexpressing cells was located in intracellular structures rather than at the cell surface (**Fig. 6B**). We also found a slight reduction in protein level and shift in intracellular distribution of βcatenin, an important mediator of the Wnt signalling and link between E-cadherin and the actin cytoskeleton at adherens junctions [31]. Whereas in control cells majority of βcatenin was localized at the plasma membrane, in matriptase overexpressing clones significant fraction of βcatenin could be also found in cytoplasmic structures (**Fig. 6D** and **Fig. S5**)**.** Taken together these results suggest that overexpression of matriptase affects cell-cell interactions in 4T1 cells, perhaps by influencing E-cadherin/βcatenin protein level and localization.

### Effects of matriptase overexpression on in vivo growth of MDA-MB-231 and 4T1 cells in an orthotopic xenograft model

The experiments described so far demonstrated that consequences of matriptase expression in breast cancer cells may be cell context dependent. Although we observed cell line-specific consequences of matriptase overexpression on cell-cell interactions and cytoskeletal organisation in 4T1 and MDA-MB-231 cells in vitro, we saw little effect on their in vitro growth (**Fig. 4D** and **Fig. 5D**). To assess the consequences of matriptase upregulation on breast cancer cell growth in a more complex in vivo microenvironment we grew the MDA-MB-231 and 4T1 cells overexpressing matriptase and the respective control cells as orthotopic xenografts after implantation into mammary fat pads of CD1 nude mice. The clones displaying most similar growth properties in vitro were selected for these in vivo experiments (Empty A and MT-SP1 B for MDA-MB-231, Empty B and MT-SP1 B for 4T1, see **Fig. 4D** and **Fig. 5D** for growth curves).

**Figure 7:**
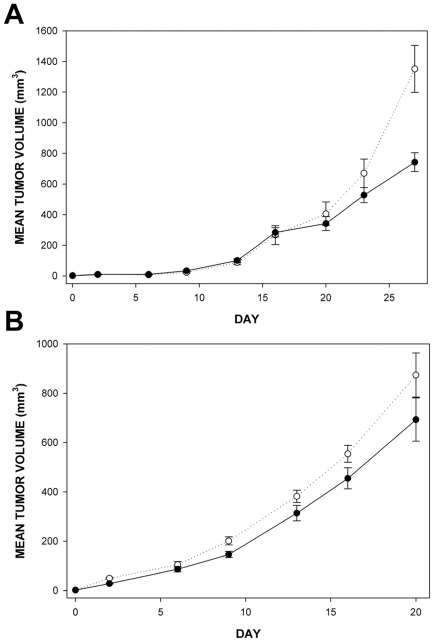
In vivo growth characteristics of indicated MDA-MB-231 and 4T1 cells engineered to stably overexpress matriptase (MT-SP1) and the respective control **clones.** The cells were injected into mammary fat pads of CD1 nude mice and grown as described in the materials and methods section. There were at least five animals in each group. (A) Growth curves of MDA-MB-231 MT-SP1 B (black circles) and MDA-MB-231 Empty A (white circles) clones. The MT-SP1 overexpressing cells grew significantly slower than the empty vector control cells (p = 0.003 on day 27). (B) Growth curves for 4T1 MT-SP1 B (black circles) and 4T1 Empty B (white circles) clones. The MT-SP1 overexpressing cells grew slower than the empty vector control cells although this was not statistically significant (p = 0.107 on day 16 and p = 0.187 on day 20). Error bars represent standard errors.

As illustrated in **Fig. 7**, in both cellular backgrounds the cells overexpressing matriptase showed decreased growth compared to the controls. This suggests that in breast cancer cells matriptase may have some growth inhibitory functions in vivo. It is worth noticing that in the case of 4T1 cells the reduction in the E-cadherin level observed in vitro (**Fig. 6B**) was also evident in vivo (**Fig. S6**).

## Discussion

Proteases constitute about 2% of all proteins encoded in human genome and they play essential roles in multiple biological processes. Over the past decades overwhelming evidence for the importance of proteases in cancer has accumulated in the literature, but the high hopes for protease targeting strategies as anticancer therapeutics has not yet fully materialised. In fact, protease inhibitors have generally failed in clinical trials indicating that more in depth understanding of the diversity and complexity of the functions of proteases in cancer is required before effective therapeutic strategies can be designed and implemented.

Matriptase is one of the proteases that attracted considerable interest of cancer biologists in recent years. There is convincing evidence linking matriptase to cancer in several systems (reviewed in [3], [14]), and matriptase is also a known activator of proteins with established roles during carcinogenesis such as hepatocyte growth factor (HGF) [11], urokinase-type plasminogen activator (uPA) [11] and matrix metalloproteinase 3 [32]. Consequently, considerable effort was invested in the development of potential matriptase-targetting therapeutics [15], [16], [17]. Despite this, the significance of matriptase in several types of human cancer remains unclear. Only a few published studies have tried to address the importance of this protein in breast cancer and they provided rather confusing results. In some studies, low matriptase protein expression was independently predictive of poorer survival (including poorer survival in node-negative group) [33], whereas in others, high matriptase level was associated with poorer survival among node-negative breast cancer cases [34]. In a further study, no association was observed between the levels of matriptase mRNA and survival [35]. Increased matriptase protein expression was reported among advanced breast cancer cases in Chinese women [36]. More recently, a coordinate overexpression of matriptase mRNA, and mRNA's for macrophage stimulating protein and macrophage stimulating protein receptor Ron was described to associate with metastasis and poor prognosis [37]. These rather conflicting data may reflect some natural variability in human populations and/or result from differences in the techniques/reagents used. They also strongly emphasise the need for additional independent studies to increase our understanding of the expression and roles of matriptase during breast carcinogenesis.

Here, we have independently assessed the significance and role of matriptase in breast cancer by evaluating protein expression levels of matriptase in established breast cancer cell lines and primary breast tumors using reverse phase protein arrays, and by studying the behaviour of breast cancer cells engineered to stably overexpress this protein.

The past studies on established cell lines demonstrated deregulation of matriptase in breast cancer cells as compared to non-transformed mammary epithelial cells [38]. In addition the functions and regulation of matriptase in breast cancer cells seem to be very complex and different from those in other cancer cell types. For example, it has been shown that matriptase activation and shedding with its cellular inhibitor HAI-1 is induced by steroid sex hormones in human prostate cancer cells, but not in breast cancer cells [39]. The activation of matriptase in breast cancer cells can be triggered by multiple events including blood derived factors and acidic pH [5], [40]. It is also likely that matriptase has protease-activity independent functions [2].

Our results show that there is considerable diversity of matriptase protein expression levels among breast cancers, with a substantial proportion of human breast cancers and established breast cancer cell lines not expressing detectable amounts of matriptase at all. The fact that significant proportion of breast cancers does not express detectable levels of matriptase may have direct consequences for further development and future use of matriptase-targeting therapies. It is unlikely that such therapies will be effective in breast cancer patients with negligible matriptase expression in the tumor. Consequently, it may be advisable to pre-screen patients for biomarkers of matriptase expression before subjecting them to therapeutic intervention in clinical trials. Interestingly the bioinformatic analysis indicated that the lack of matriptase expression in breast cancers may be associated with pre-translational rather than post-translational mechanisms. This conclusion is supported by recent finding that elimination of matriptase expression in MDA-MB-231 cells may be due to the cleavage of its mRNA by miR-27b microRNA [41].

The observation that stable overexpression of matriptase in two breast cancer cell lines was associated with diverse phenotypical outcomes indicates that the actions of matriptase may be fine-tuned for particular cellular context rather than follow universally applicable patterns. This may reflect the fact that cellular regulation and functions of matriptase are extremely complex and still poorly understood [2]. For example, it has been reported that HER2 signalling via the phosphatidylinositol 3-kinase pathway results in increased matriptase zymogen activity in prostate cancer cells [42]. Our finding that matriptase expression is correlated with HER2 status supports the notion that matriptase function may be linked to HER2 signalling in cancer cells. Another example of dependence of matriptase function on other cellular signalling cascades is provided in the recent work of Szabo et al., who demonstrated that genetic ablation of hepatocyte growth factor receptor c-Met completely negates the oncogenic potential of matriptase in matriptase expressing keratinocytes [43]. It is interesting to notice that in 4T1 cells overexpression of matriptase was associated with reduction in cell-cell contacts and downregulation of a major cell adhesion protein E-cadherin. The role of matriptase in regulation of E-cadherin has been recently proposed in Mardin-Darby canine kidney (MDCK) cells although the precise mechanism of this regulation remains uncertain [44]. There is also accumulating evidence for the involvement of matriptase in epithelial-mesenchymal transition (EMT) [44], [45]. EMT is believed to play important role in tumor spread and dawnregulation of E-cadherin associated with reduction in cell-cell interactions represents one of the hallmarks of this process. Our data in 4T1 cells are certainly in agreement with those scenarios suggesting that matriptase may play a role in breast cancer spread and metastasis. This idea finds additional support in the fact that knock down of matriptase in 4T1 cells has been reported to be associated with decreased metastatic potential [46]. Although 4T1 cells represent a mouse cell line and there is no certainty that the results obtained using this model can be extrapolated to human cells, it is worth noticing, that published evidence exists that links matriptase to regulation of cell adhesion in human cells. Importantly, matriptase signalling has been implicated in a rounded phenotype similar to that we observed in 4T1 cells in a human breast cancer cell line [8]. On the other side it is noteworthy that in both MDA-MB-231 and 4T1 cells matriptase overexpression was associated with moderately inhibited tumor growth in vivo. These results contrast with growth promoting actions of matriptase suggested in some other tumor types [15], [32], and indicate that in breast cancer matriptase may display both tumor promoting and tumor suppressing activities.

## Materials and Methods

### Ethics statement

The study was approved by the Lothian Research Ethics Committee (08/S1101/41). No informed consent (written or verbal) was obtained for use of retrospective tissue samples from the patients within this study, most of whom were deceased, since this was not deemed necessary by the Ethics Committee, who waived the need for consent.

All procedures involving animals were carried out in accordance with UK Coordinating Committee on Cancer Research guidelines by approved protocol (Home Office Project Licence no. 60/3576).

### Protein extraction from frozen tissue and cell lines

Protein was extracted from 107 breast tumours treated within the Edinburgh Breast Unit, all of which had pathological confirmation of malignancy. Tumor material was placed in an ice-cold flat bottomed soda-glass tube (50×12mm) with 0.3 ml of Lysis buffer (50 mM Tris pH 7.5; 5 mM EGTA pH 8.5; 150 mM NaCl supplemented with protease inhibitors (Roche 11836153001), phosphatase inhibitors (Sigma P2850; P5726) and aprotinin (Sigma A6279)). Samples were homogenised on ice at full power for 2×10sec (with a 30 sec interval between bursts to allow the sample to cool down) using a Silverson homogeniser. Resulting homogenates were transferred to pre-cooled microcentrifuge tubes and residual material recovered from the homogeniser with a further 2×0.3 ml of lysis buffer (total pooled volume of each sample = 0.9 ml). Triton X-100 was added to each sample (9 µl/0.9 ml) before centrifuging at 13,000 g for 30 min at 4°C after which supernatants were transferred to fresh microcentrifuge tubes. Total protein concentrations were determined by BCA assay (Thermo Scientific, #23235) and normalised at 2 mg/ml. The cell line lysates were prepared in a similar way using the same lysis buffer.

### Reverse phase protein arrays (RPPA)

Denatured and reduced protein lysates were spotted onto LI-COR (LI-COR Biosciences, Nebraska, USA) nitrocellulose-coated glass slides as previously described [47]. Three replicates were spotted per sample in five two-fold dilutions. Slides were hydrated in Li-Cor blocking buffer for 1 hour (LI-COR Biosciences, Nebraska, USA), and then incubated with previously optimised primary antibodies overnight at 4°C in a sealed box containing a damp paper towel. The following day slides were washed in PBS/T at room temperature for 5 minutes (×3) before incubating with far-red fluorescently-labelled secondary antibodies diluted in Li-Cor Odyssey Blocking Buffer (1 µl/2 ml) at room temperature for 45 mins with gentle shaking. Slides were then washed in excess PBS/T (×3)/PBS (×3) and allowed to air dry before reading on a Li-Cor Odyssey scanner at 680 nm and 780 nm.

RPPA analysis was performed using MicroVigene RPPA analysis module (VigeneTech, Carlisle, MA, USA). Spots were quantified by accurate single segmentation, with actual spots signal boundaries determined by the image analysis algorithm. Each spot intensity was quantified by measuring the total pixel intensity of the area of each spot (volume of spot signal pixels), with background subtraction of 2 pixels around each individual spot. The mean of the replicates was used for normalization and curve fitting. Curve fitting was performed using four parameter logistical non-linear regression using a joint estimation approach.

### Bioinformatics

Expression data from Neve et al. [23] was downloaded from Array Express (E-TABM-157) and expression values derived using the RMA algorithm (Bioconductor affy package) combined with an updated microarray annotation (U133A, Ensembl gene CDF version 11 [24]).

### Plasmid vectors

pHygpβactin-EcoRV-IRES-mCherry plasmid was created by introducing a cassette containing chicken βactin promoter, EcoRV restriction site, internal ribosome entry site and mCherry coding sequence into the pTKHyg plasmid backbone (Clontech). The pHygpβactin-Matriptase-IRES-mCherry plasmid was subsequently generated by introducing a PCR amplified human matriptase open reading frame into the EcoRV site of pHygpβactin-EcoRV-IRES-mCherry vector. pHygpβactin-Matriptase-IRES-mCherry and pHygpβactin-EcoRV-IRES-mCherry plasmids were used to generate MT-SP1 and Empty clones, respectively.

### Cell culture, transfections, and selection of stable cell lines

The MDA-MB-231 [25], 4T1 [26] and other cells [27] were grown in DMEM (Gibco/Invitrogen). The medium was supplemented with 10% fetal calf serum, 100 U/ml penicillin and 100µg/ml streptomycin. Transfections were performed using Amaxa Nucleofector technology (Lonza) accordingly to the manufacturer's protocols. Stable cell lines were selected using previously described protocol [48]. Hygromycin B (Calbiochem, cat. no. 400052) at 250 µg/ml was used as the selection agent and mCherry signal was utilised for flow cytometric sorting of positive cells after selection.

### Protein precipitation from the conditioned medium

To analyse the presence of matriptase in the conditioned media of engineered MDA-MB-231 and 4T1 cells, the cells were seeded on day 0 into 10 cm tissue culture dishes (TPP, cat. no. 93100) to reach ∼90% confluency the next day (day 1). On day 1 the cells were rinsed with 10 ml of serum free DMEM and overlayed with 5 ml of serum free Optimem with Glutamax (Gibco/Invitrogen cat. no. 51985). After 24 h the medium was transferred into 15 ml falcon tube and spun down 3 min at 1100 RPM to pellet any potential floating cells. The supernatant was transferred into 50 ml falcon tube and precipitated using 36 ml of 100% ethanol at –20°C for 48 h. Subsequently the resulted precipitate was pulled down by centrifugation for 1h at 4600 RPM (4°C) in the Sorvall Legend RT centrifuge. The supernatant was decanted and the pellet rinsed with 20 ml of 90% ethanol (−20°C). This was followed by centrifugation for 30 min at 4600RPM. The supernatant was removed and the pellet dried. Subsequently 200 µl of 3xSDS-Samble Buffer + β mercaptoethanol was added to extract the pelleted proteins. After short incubation (∼5 min) the samples were transferred to 1.5 ml eppendorf tubes and incubated for 3 min at 100°C. They were subsequently frozen and stored at −20°C until SDS-PAGE and western blotting. 20 µl of each sample was loaded pro lane of 10% gel.

### Antibodies, Gel Electrophoresis and Western Blot Analysis

SDS-PAGE and western blotting were performed as previously described [49]. The following primary antibodies were used: rabbit polyclonal anti matriptase/ST14 (Bethyl Laboratories, cat. no. A300–221A), rabbit monoclonal anti Src (Cell Signaling, cat. no. 2109), rabbit polyclonal anti phospho-Src (Tyr416) (Cell Signaling, cat. no. 2101), mouse monoclonal anti FAK (Upstate biotechnology, cat. no. 05–537), rabbit polyclonal anti phosphor-FAK (Tyr397) (Invitrogen cat. no. 44624G), mouse monoclonal anti E-cadherin (BD Biosciences, cat. no. 610182), mouse monoclonal anti β-catenin (BD Transduction Laboratories, cat. no. 610154), polyclonal rabbit anti γ-tubulin (Sigma-Aldrich, cat. no. T3559).

### In vitro growth assay

To assess in vitro proliferation the cells were plated out at 1×10^5^ cells/4ml of medium per well in six-well plates (Costar 3516, Corning). They were subsequently counted every day for a period of six days. The medium from the well was collected, the adherent cells were trypsinized using 1ml of 0.05% trypsin solution and added to the medium collected from the well. The cells were spun down for 5 min at 1100 RPM in a standard table top centrifuge, resuspended in PBS (250 µl day 1, 500 µl day 2, 1 ml day 3, 3 ml day 4, 5 ml days 5 and 6) and counted using haemocytometer. Three independent experiments (each consisting of three independent repeats counted in duplicates) were performed and the data from a representative experiment are displayed. Potential differences between different clones at different time points were analysed using series of student's T-tests.

### Cell detachment assay

For cell detachment assay 3×10^6^ cells/well were seeded in 6well plates (Corning) in 4 ml medium. After 20 h the cells were washed twice with 1.5 ml of PBS and 1.5 ml of PBS/EDTA was added. The cells were subjected to mechanical stress by rotatory shaking (level 6 on the Heidolph rotomax 120 shaker). After 15min the detached cells were collected by transfering PBS/EDTA containing detached cells into a universal tube containing 3 ml of medium. The attached cells were trypsynized using 1 ml of 0.05% trypsin solution and transferred into a separate universal tube containing 3 ml of medium. Both the detached cells and the attached cells were spun down for 5 min at 1100 RPM in a standard table top centrifuge and their numbers estimated using haemocytometer after resuspension in 500 µl of PBS. The presented data are based on three independent experiments each containing two repeats counted in duplicates. The differences between the groups were analysed using student's T-tests.

### Transwell migration assay

For transwell cell migration assays 1×10^5^ cells in 100 µl DMEM containing 1%FCS were placed in the top part of the 6.5 mm diameter, 8.0 µm pore size transwell chamber (Corning, Lowell, MA, USA cat. no. 3422), the bottom part was filled with 600 µl of DMEM containing 10%FCS. The cells were allowed to migrate for 16 hrs. They were subsequently fixed in ice cold 100% methanol and the nuclei of cells that migrated through the membrane were stained with propidium iodide (10 µg/ml in PBS). The number of migrated cells was estimated by counting the cells in 6 randomly selected fields of view using fluorescent microscope (40× objective). The presented data are based on three independent experiments each containing two repeats. The differences between the groups were analysed using student's T-tests.

### Immunofluorescence

For immunofluorescence studies the cells were seeded on glass coverslips in 12 well tissue culture plates (TPP, cat. no. 92012). After 24–30 hrs the cells were rinsed twice with PBS, fixed with 3.7% formaldehyde and stained with appropriate antibodies using previously described procedures [50]. The same anti E-cadherin, β-catenin, FAK and phospho-Src antibodies as for western blotting were used. The secondary antibodies were Alexa Fluor 488 labeled anti mouse (Invitrogen, cat. no. A11029) or anti rabbit (Invitrogen, cat. no. A11034) immunoglobulines. After staining the coverslips were mounted on microscopic slides using Vectashield with DAPI (Vector Laboratories cat. no. H-1200) and images were acquired with FV1000 confocal microscope and 63x objective as previously described [49].

### Orthotopic tumor xenografts

For in vivo growth analysis 1×10^6^ cells in 50 µl of 1∶1 HBSS/matrigel mixture (Gibco cat no. 14025/BD Biosciences cat. no. 354234) were injected into the mammary fat pads of 6–8 week-old female CD1 nude mice (Charles River). Mice were housed in an individually ventilated caging system on a 12-hour light/dark environment maintained at constant temperature and humidity. Tumor measurements were performed twice a week using a calliper. Tumor volume was defined as length x width^2^/2. There were seven animals in each empty vector control and MT-SP1 overexpressing group for MDA-MB-231 cells and five animals per group for 4T1 cells. Student's T-tests were used to statistically evaluate the data.

## Supporting Information

Figure S1 **Analysis of matriptase (MT-SP1) protein levels in indicated cell lines determined using standard western blotting approach.** Tubulin and Ponceau stainings were performed as loading controls.
(PDF)

Figure S2 **Comparison of in vitro migratory properties (A) and attachment strength (B) in the** **indicated MDA-MB-231 clones and the parental cell line.** (See materials and methods for details.). No statistically significant differences between the clones were found with respect to in vitro migratory properties (p>0.05). Although some “between-clone” variations were found (p<0.05) with respect to attachment strength, they were not associated with the presence or absence of MT-SP1 overexpression. Error bars represent standard errors.
(PDF)

Figure S3 **Matriptase (MT-SP1) protein levels in the 4T1 MT-SP1 A cells and in the subpopulation of these cells that spontaneously reverted t**o **a “flat” morphology.** (A) Bright field image of the initial 4T1 MT-SP1 A cells. (B) Bright field image of the “flat” morphology subpopulation established from 4T1 MT-SP1 A clone. (C) Western blot showing matriptase levels in the initial 4T1 MT-SP1 A cells (Initial) and in 4T1 MT-SP1 A cells that reverted to a “flat” phenotype (Reverted). Actin represents the loading control. Note that we purposefully overloaded the “Reverted” line to underline the decrease in matriptase level. The 4T1 MT-SP1 A cells that reverted to a “flat” phenotype were established after multiple (>20) passages. They were split using 0.05% trypsin solution in PBS after a short wash (∼1 min) with low concentration (0.025%) trypsin in PBS to remove less adherent cells. The cells were split 1∶16 every three days.
(PDF)

Figure S4 **Focal adhesion kinase and c-Src in 4T1 cells with stable matriptase (MT-SP1) overexpression and control cells.** (**A**) Western blots illustrating total Src and P-Src(Y416) levels in the indicated cell lines (left), and representative immunofluorescence pictures of P-Src staining (green) in MT-SP1 overexpressing cells and control cells (right). (**B**) Western blots illustrating total FAK and P-FAK(Y397) levels in the indicated cell lines (left), and representative immunofluorescence pictures of FAK staining (green) in MT-SP1 overexpressing cells and control cells (right). The immunofluorescence data are for clones 4T1 Empty B and 4T1 MT-SP1 B respectively, but analogous results were obtained in 4T1 Empty A and 4T1 MT-SP1 A clones. Blue color represents DAPI staining (nuclei). Scale bars 10 µm.
(PDF)

Figure S5 **Individual images for β-catenin (top) and DAPI (bottom) stainings in 4T1 cells overexpressing MT-SP1 (right) or control cells transfected with empty vector (left).** The same images are presented as overlays in Fig. 6D. Scale bar 30 µm.
(PDF)

Figure S6 **Immunohistochemistry for E-cadherin in tumor sections derived from 4T1 Empty (A), and 4T1 MT-SP1 (B), orthotopic xenografts. (C) and (D) represent “no primary** **antibody” controls for (A) and (B) respectively.** Representative pictures were selected. The details of the immunohistochemistry are provided in the “Immunohistochemistry protocol” section of this figure.
(PDF)

Table S1 **Clinical data associated with the set of 107 primary tumor samples used in this study.**
(PDF)
