# Supplementary material for: Diversity of Matriptase Expression Level and Function in Breast Cancer
Source: PLoS One. 2012 Apr 13;7(4):e34182. doi: 10.1371/journal.pone.0034182 (PMC3325989; doi:10.1371/journal.pone.0034182)
Supplement: Figure S1 — Analysis of matriptase (MT-SP1) protein levels in indicated cell lines determined using standard western blotting approach. Tubulin and Ponceau stainings were performed as loading controls. (PDF) [file pone.0034182.s001.pdf]

Figure S1

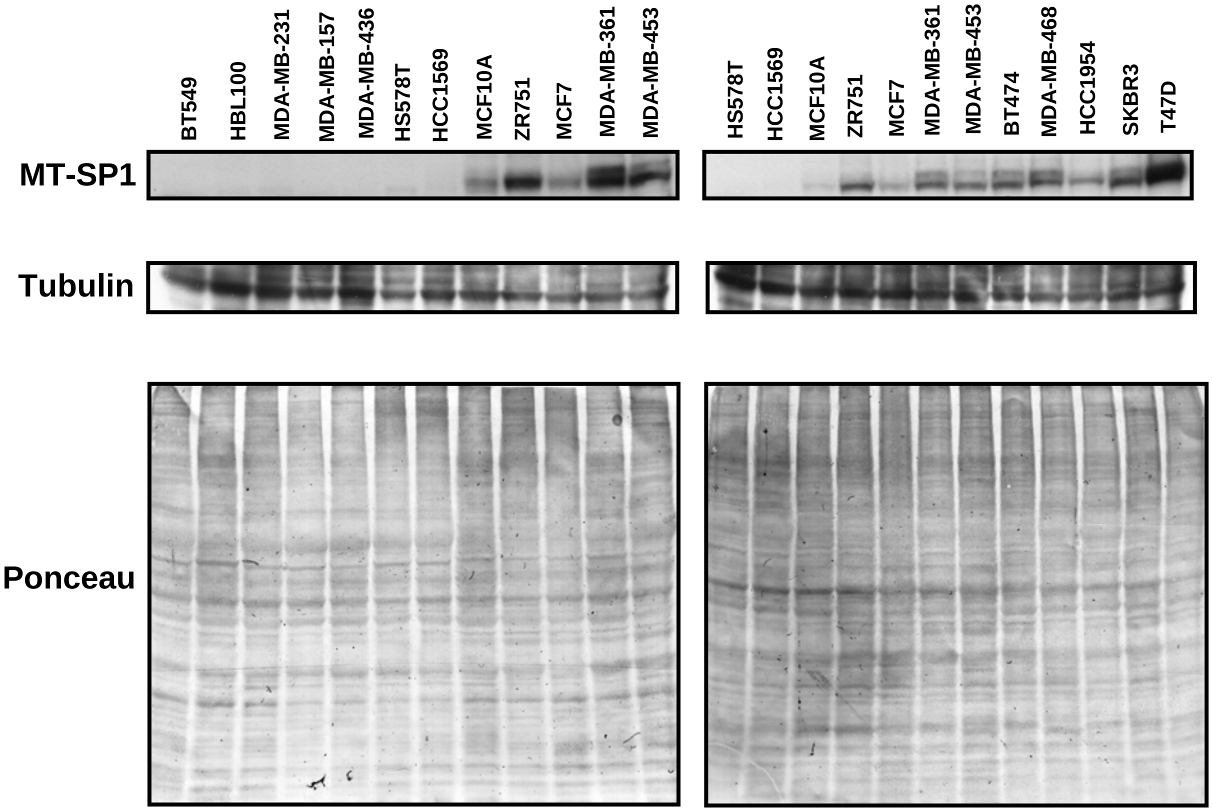

Figure S1: Analysis of matriptase (MT-SP1) protein levels in indicated cell lines determined using standard western blotting approach. Tubulin and Ponceau stainings were performed as loading controls.
