# Supplementary material for: Diversity of Matriptase Expression Level and Function in Breast Cancer
Source: PLoS One. 2012 Apr 13;7(4):e34182. doi: 10.1371/journal.pone.0034182 (PMC3325989; doi:10.1371/journal.pone.0034182)
Supplement: Figure S2 — Comparison of in vitro migratory properties (A) and attachment strength (B) in the indicated MDA-MB-231 clones and the parental cell line. (See materials and methods for details.). No statistically significant differences between the clones were found with respect to in vitro migratory properties (p>0.05). Although some “between-clone” variations were found (p<0.05) with respect to attachment strength, they were not associated with the presence or absence of MT-SP1 overexpression. Error bars represent standard errors. (PDF) [file pone.0034182.s002.pdf]

Figure S2 :

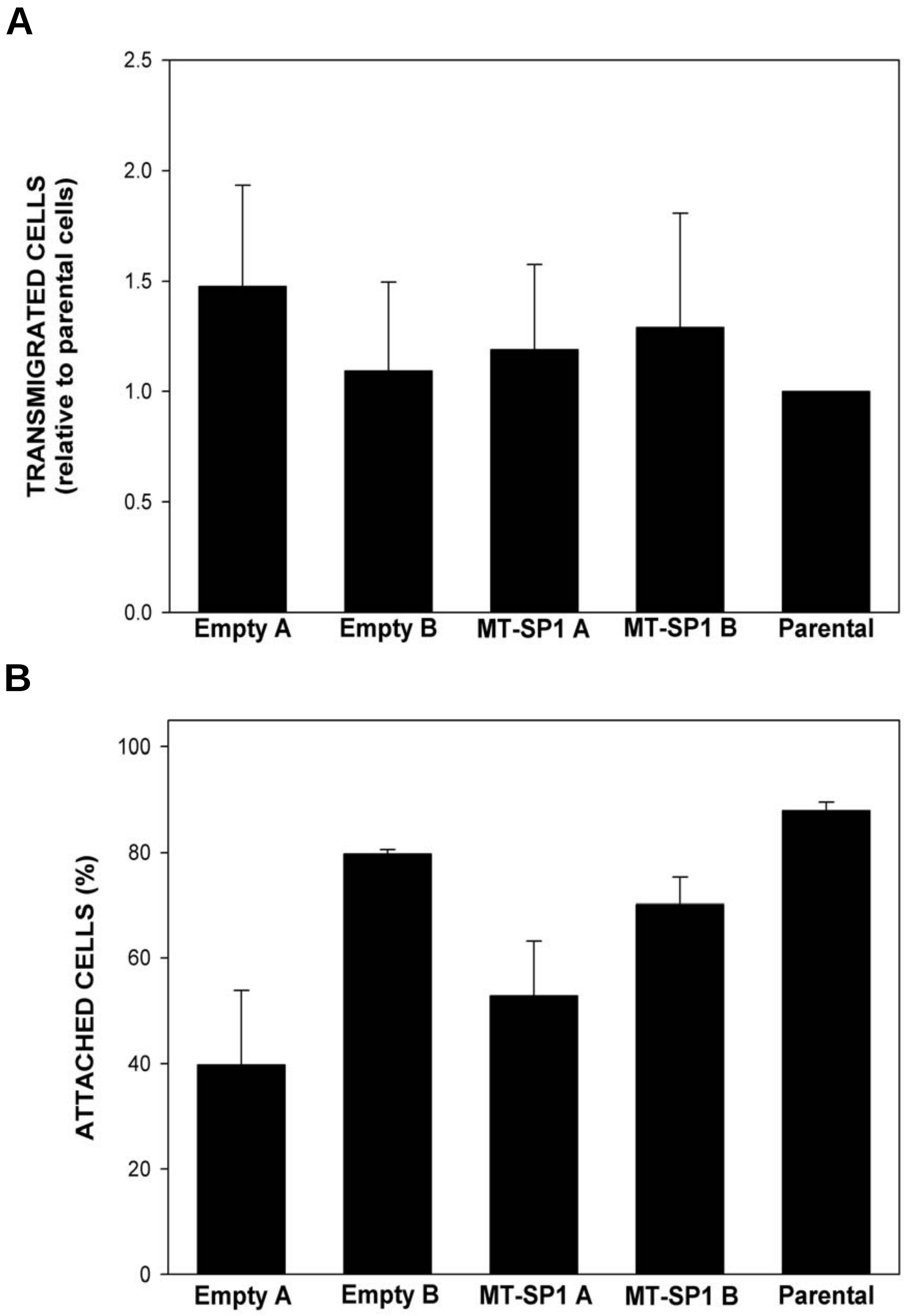

Figure 2S: Comparison of in vitro migratory properties **(A)** and attachment strength **(B)** in the indicated MDA-MB-231 clones and the parental cell line. (See materials and methods for details.). No statistically significant differences between the clones were found with respect to in vitro migratory properties ($p>0.05$). Although some “between-clone” variations were found ($p<0.05$) with respect to attachment strength, they were not associated with the presence or absence of MT-SP1 overexpression. Error bars represent standard errors.
