# Supplementary material for: Diversity of Matriptase Expression Level and Function in Breast Cancer
Source: PLoS One. 2012 Apr 13;7(4):e34182. doi: 10.1371/journal.pone.0034182 (PMC3325989; doi:10.1371/journal.pone.0034182)
Supplement: Figure S3 — Matriptase (MT-SP1) protein levels in the 4T1 MT-SP1 A cells and in the subpopulation of these cells that spontaneously reverted to a “flat” morphology. (A) Bright field image of the initial 4T1 MT-SP1 A cells. (B) Bright field image of the “flat” morphology subpopulation established from 4T1 MT-SP1 A clone. (C) Western blot showing matriptase levels in the initial 4T1 MT-SP1 A cells (Initial) and in 4T1 MT-SP1 A cells that reverted to a “flat” phenotype (Reverted). Actin represents the loading control. Note that we purposefully overloaded the “Reverted” line to underline the decrease in matriptase level. The 4T1 MT-SP1 A cells that reverted to a “flat” phenotype were established after multiple (>20) passages. They were split using 0.05% trypsin solution in PBS after a short wash (∼1 min) with low concentration (0.025%) trypsin in PBS to remove less adherent cells. The cells were split 1∶16 every three days. (PDF) [file pone.0034182.s003.pdf]

Figure S3

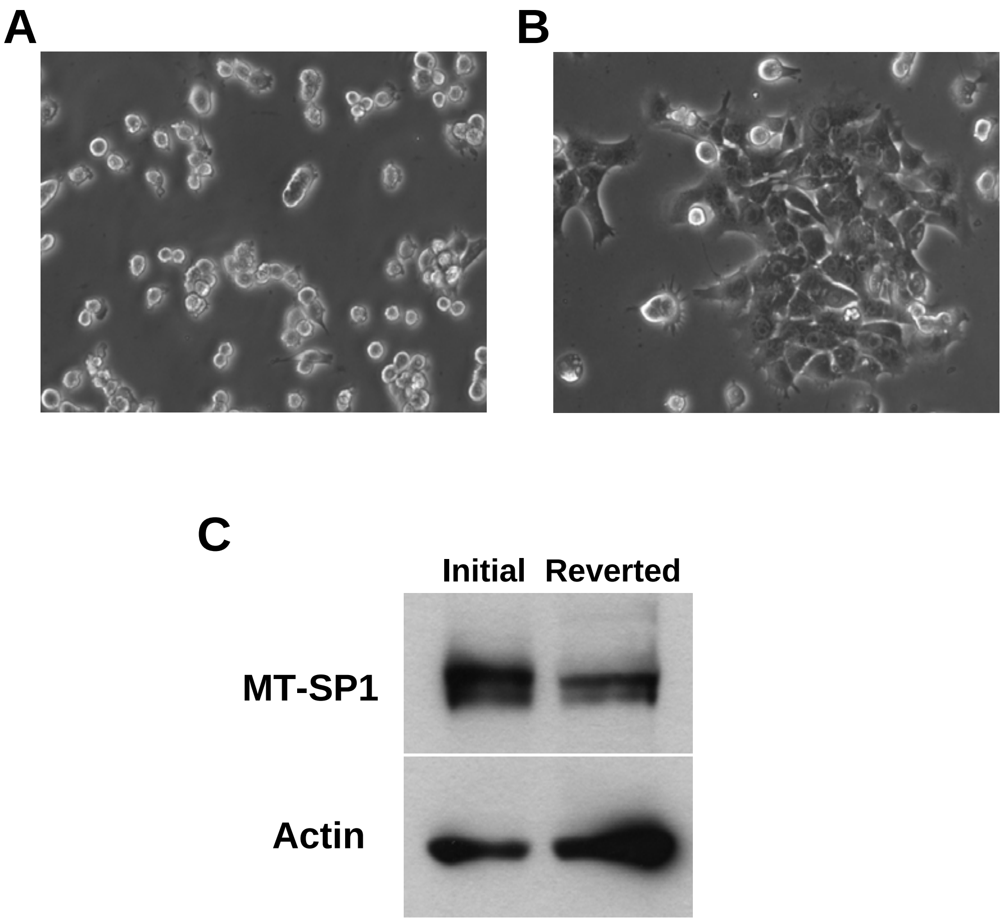

Figure S3: Matriptase (MT-SP1) protein levels in the 4T1 MT-SP1 A cells and in the subpopulation of these cells that spontaneously reverted to a “flat” morphology. **(A)** Bright field image of the initial 4T1 MT-SP1 A cells. **(B)** Bright field image of the “flat” morphology subpopulation established from 4T1 MT-SP1 A clone. (C) Western blot showing matriptase levels in the initial 4T1 MT-SP1 A cells (Initial) and in 4T1 MT-SP1 A cells that reverted to a “flat” phenotype (Reverted). Actin represents the loading control. Note that we purposefully overloaded the “Reverted” line to underline the decrease in matriptase level. The 4T1 MT-SP1 A cells that reverted to a “flat” phenotype were established after multiple (>20) passages. They were split using 0.05% trypsin solution in PBS after a short wash (~1min) with low concentration (0.025%) trypsin in PBS to remove less adherent cells. The cells were split 1:16 every three days.
