# Supplementary material for: Diversity of Matriptase Expression Level and Function in Breast Cancer
Source: PLoS One. 2012 Apr 13;7(4):e34182. doi: 10.1371/journal.pone.0034182 (PMC3325989; doi:10.1371/journal.pone.0034182)
Supplement: Figure S4 — Focal adhesion kinase and c-Src in 4T1 cells with stable matriptase (MT-SP1) overexpression and control cells. (A) Western blots illustrating total Src and P-Src(Y416) levels in the indicated cell lines (left), and representative immunofluorescence pictures of P-Src staining (green) in MT-SP1 overexpressing cells and control cells (right). (B) Western blots illustrating total FAK and P-FAK(Y397) levels in the indicated cell lines (left), and representative immunofluorescence pictures of FAK staining (green) in MT-SP1 overexpressing cells and control cells (right). The immunofluorescence data are for clones 4T1 Empty B and 4T1 MT-SP1 B respectively, but analogous results were obtained in 4T1 Empty A and 4T1 MT-SP1 A clones. Blue color represents DAPI staining (nuclei). Scale bars 10 µm. (PDF) [file pone.0034182.s004.pdf]

Figure S4

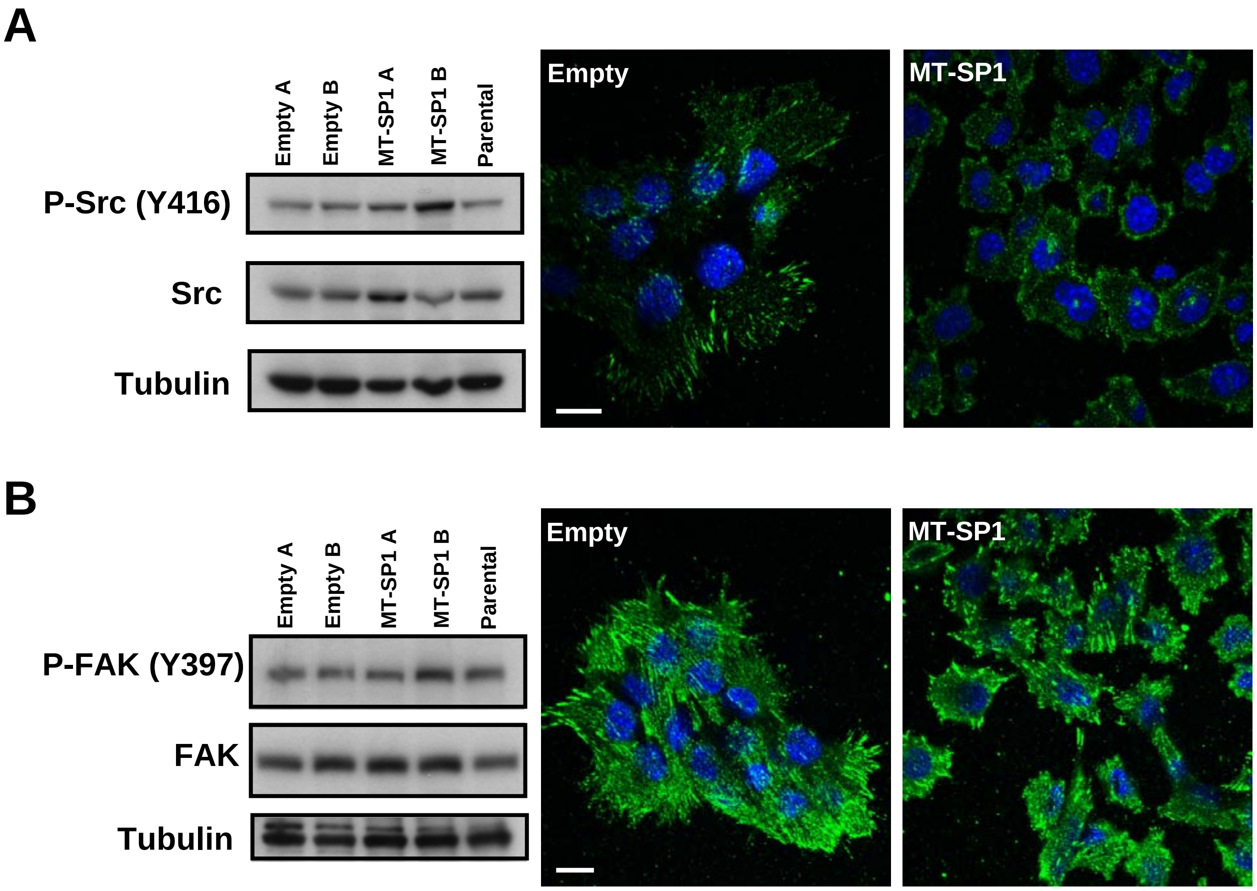

Figure S4: Focal adhesion kinase and c-Src in 4T1 cells with stable matriptase (MT-SP1) overexpression and control cells. **(A)** Western blots illustrating total Src and P-Src(Y416) levels in the indicated cell lines (left), and representative immunofluorescence pictures of P-Src staining (green) in MT-SP1 overexpressing cells and control cells (right). **(B)** Western blots illustrating total FAK and P-FAK(Y397) levels in the indicated cell lines (left), and representative immunofluorescence pictures of FAK staining (green) in MT-SP1 overexpressing cells and control cells (right). The immunofluorescence data are for clones 4T1 Empty B and 4T1 MT-SP1 B respectively, but analogous results were obtained in 4T1 Empty A and 4T1 MT-SP1 A clones. Blue color represents DAPI staining (nuclei). Scale bars 10μM.
