# Supplementary material for: Diversity of Matriptase Expression Level and Function in Breast Cancer
Source: PLoS One. 2012 Apr 13;7(4):e34182. doi: 10.1371/journal.pone.0034182 (PMC3325989; doi:10.1371/journal.pone.0034182)
Supplement: Figure S5 — Individual images for β-catenin (top) and DAPI (bottom) stainings in 4T1 cells overexpressing MT-SP1 (right) or control cells transfected with empty vector (left). The same images are presented as overlays in Fig. 6D. Scale bar 30 µm. (PDF) [file pone.0034182.s005.pdf]

Figure S5

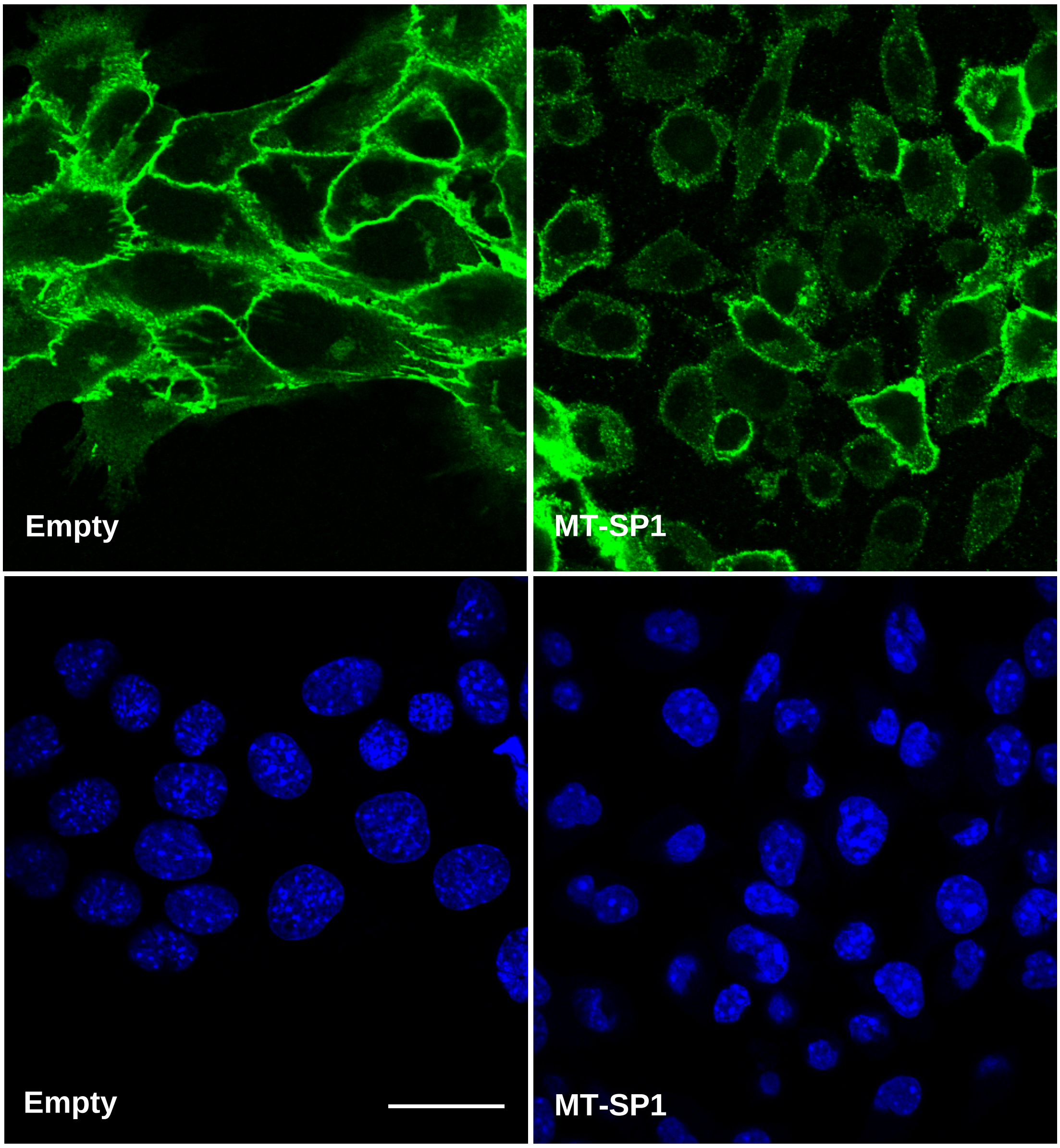

Figure S5: Individual images for β-catenin (top) and DAPI (bottom) stainings in 4T1 cells overexpressing MT-SP1 (right) or control cells transfected with empty vector (left). The same images are presented as overlays in Figure 6D. Scale bar 30μM.
