# Supplementary material for: Diversity of Matriptase Expression Level and Function in Breast Cancer
Source: PLoS One. 2012 Apr 13;7(4):e34182. doi: 10.1371/journal.pone.0034182 (PMC3325989; doi:10.1371/journal.pone.0034182)
Supplement: Figure S6 — Immunohistochemistry for E-cadherin in tumor sections derived from 4T1 Empty (A), and 4T1 MT-SP1 (B), orthotopic xenografts. (C) and (D) represent “no primary antibody” controls for (A) and (B) respectively. Representative pictures were selected. The details of the immunohistochemistry are provided in the “Immunohistochemistry protocol” section of this figure. (PDF) [file pone.0034182.s006.pdf]

# Figure S6

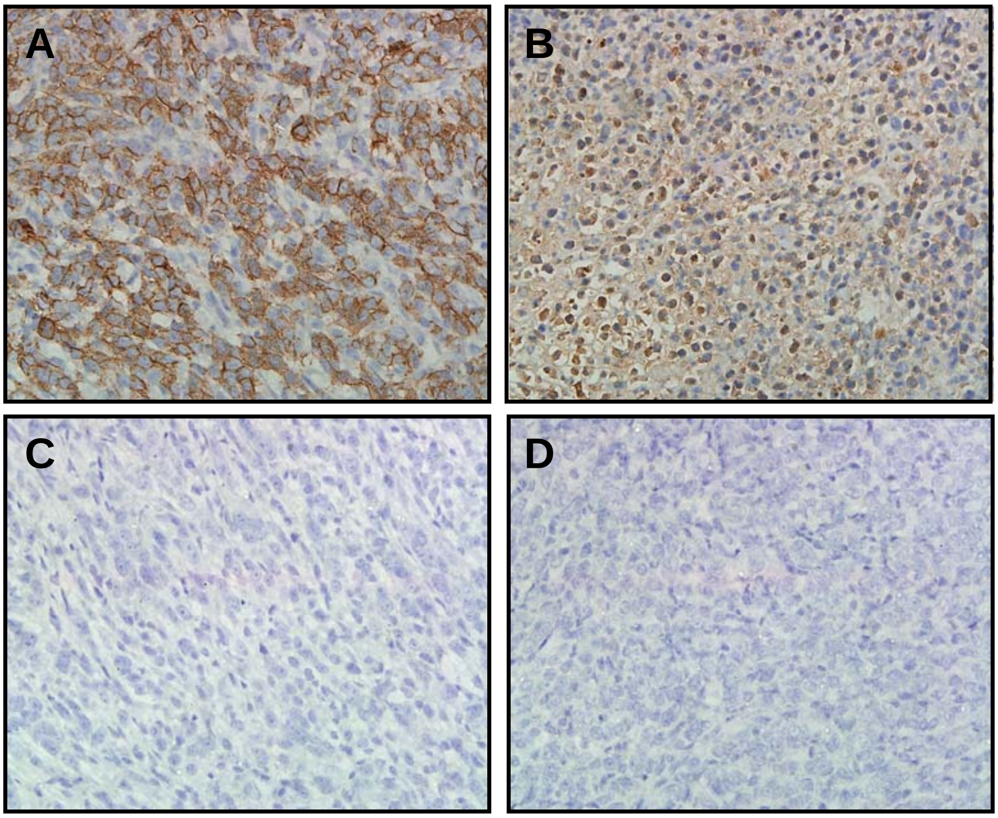


**Figure S6:** Immunohistochemistry for E-cadherin in tumor sections derived from 4T1 Empty **(A)**, and 4T1 MT-SP1 **(B)**, orthotopic xenografts. **(C)** and **(D)** represent "no primary antibody" controls for (A) and (B) respectively. Representative pictures were selected. The details of the immunohistochemistry are provided in the "Immunohistochemistry protocol" section of this figure.

## Immunohistochemistry Protocol

For E-cadherin immunohistochemistry tumor samples were fixed, embedded in paraffin blocks, microtome sectioned, and mounted onto microscope slides as described in detail previously [Barraclough J, Hodgkinson C, Hogg A, Dive C, Welman A (2007) Increases in c-Yes expression level and activity promote motility but not proliferation of human colorectal carcinoma cells. Neoplasia 9: 745-754.].

Subsequently the samples were processed as follows:

1. Dewax in xylene 2x5mins.
2. Rehydration 5mins in 99%, 99%, 80% and 50% ethanol.
3. Boil sections in pressure cooker containing antigen retrieval buffer for 10min (10mM Sodium Citrate pH 6.0)
4. Leave to cool for 20min.
5. Wash sections 2x5 min in 1xPBS.
6. Draw around tissue with Immedge pen.
7. Treat sections with Peroxidase block (from DAKO Kit K4011) or 3% H2O2 for 10 mins.
8. Wash slides with PBST for 5 min.
9. Block with DAKO Total protein block (DAKO X0909) for 10 min.
10. Dilute primary antibody (Rabbit monoclonal anti E-Cadherin, Cell Signalling cat. no. 4065) 1:500 in DAKO Antibody dilutent (DAKO S0809).
11. Incubate for 1hour at room temperature or overnight at 4°C.
12. Wash sections 2x PBST for 5 min.
13. Incubate section with DAKO Envision labelled polymer (Rabbit) for 30mins.
14. Wash slides with PBST 2x 5 min.
15. Add DAB/DAB Chromogen for 10 min (DAKO K4011)
16. Wash sections in water.
17. Counterstain in Heamatoxylin (Sigma MHS32-1L) for approx. 1 min and Scot's tap water for approx.1 min.
18. Dehyration 5 mins in 50%, 80%, 99% and 99% ethanol.
19. Mounting with DPX (Fisher D/5319/05).
