# Supplementary material for: Diversity of Matriptase Expression Level and Function in Breast Cancer
Source: PLoS One. 2012 Apr 13;7(4):e34182. doi: 10.1371/journal.pone.0034182 (PMC3325989; doi:10.1371/journal.pone.0034182)
Supplement: Table S1 — Clinical data associated with the set of 107 primary tumor samples used in this study. (PDF) [file pone.0034182.s007.pdf]

Table S1

Cohort variable	Number	Percentage
**Histological Type**		
No special type (NST)	89	83.2
Lobular / pleomorphic lobular	9	8.4
Tubular / tubulolobular	5	4.7
Other	4	3.7
**Grade**		
1	26	24.3
2	41	38.3
3	40	37.4
**Tumour Stage**		
1	52	48.6
2	40	37.4
3	4	3.7
4	3	2.8
NK	8	7.5
**Node status**		
Negative	65	60.7
Positive	32	29.9
NK	10	9.3
**Molecular Phenotype**		
ER+	71	66.4
Triple negative	14	13.1
HER2+	22	20.6

Table S1: Clinical data associated with the set of 107 primary tumor samples used in this study.
